# Absence of Association between *CCR5* rs333 Polymorphism and Childhood Acute Lymphoblastic Leukemia

**DOI:** 10.1155/2014/924030

**Published:** 2014-04-13

**Authors:** Carlos Eduardo Coral de Oliveira, Marla Karine Amarante, Aparecida de Lourdes Perim, Patricia Midori Murobushi Ozawa, Carlos Hiroki, Glauco Akelinghton Freire Vitiello, Roberta Losi Guembarovski, Maria Angelica Ehara Watanabe

**Affiliations:** ^1^Laboratory of Study and Application of DNA Polymorphisms, Department of Pathological Sciences, Biological Sciences Center, State University of Londrina, Rodovia Celso Garcia Cid, (PR 445), Km 380, 86051-970 Londrina, PR, Brazil; ^2^Laboratory of Hematology, Department of Pathology, Clinical and Toxicological Analysis, Health Sciences Center, State University of Londrina, Londrina, PR, Brazil

## Abstract

Acute lymphoblastic leukemia (ALL) is a malignant disorder that originates from one single hematopoietic precursor committed to B- or T-cell lineage. Ordinarily, these cells express CCR5 chemokine receptor, which directs the immune response to a cellular pattern and is involved in cancer pathobiology. The genetic rs333 polymorphism of CCR5 (Δ32), results in a diminished receptor expression, thus leading to impaired cell trafficking. The objective of the present study was to investigate the effect of *CCR5* chemokine receptor rs333 polymorphism in the pathogenesis of ALL. The genotype distribution was studied in 79 patients and compared with 80 control subjects, in a childhood population of Southern Brazil. Genotyping was performed using DNA samples amplified by polymerase chain reaction with sequence-specific primers (PCR-SSP). The homozygous (Δ32/Δ32) deletion was not observed in any subject involved in the study. Heterozygous genotype was not associated with ALL risk (OR 0.7%; 95% CI 0.21–2.32; *P* > 0.05), nor recurrence status of ALL (OR 0.86; 95% CI 0.13–5.48; *P* > 0.05). This work demonstrated, for the first time, no significant differences in the frequency of the CCR5/Δ32 genotype between ALL and control groups, indicating no effect of this genetic variant on the ALL susceptibility and recurrence risk.

## 1. Introduction

Leukemia is the most common childhood cancer, although overall incidence is rare. Within this population, acute lymphoblastic leukemia (ALL) occurs approximately five times more frequently than acute myelogenous leukemia (AML) and accounts for approximately 78% of all childhood leukemia diagnoses [1]. In Brazil, the National Cancer Institute (INCA) estimated 9,370 new cases of leukemia in 2014, with the highest age incidence of 1–4 years [2].

The specific biological and molecular mechanisms that account for the aggressiveness and poor therapy response of some ALL cases remain to be elucidated. Once chemokines and their receptors have been discovered as essential and selective mediators in leukocyte migration to inflammatory sites and to secondary lymphoid organs, it is reasonable that they also play a critical role in tumor initiation, promotion, and progression [3, 4]. Moreover, updated research indicates that cancer cells subvert the normal chemokine system, and these molecules and their receptors become important constituents of the tumor microenvironment with very different ways to exert tumor-promoting roles [5].

The CC chemokine receptor 5 (CCR5) belongs to the trimeric guanine nucleotide-binding-protein-coupled seven-transmembrane receptor superfamily, which comprises the largest superfamily of human proteins [6]. It exerts its activity via G protein and binds to the chemokines RANTES (CCL5), MIP-1*α* (CCL3), and MIP-1*β* (CCL4) [7]. This receptor is involved in the chemotaxis of leucocytes to inflammation sites [8] and plays important function in the recruitment of macrophages, T cells, and monocytes [9].

The importance of CCR5 for immune response is dependent on the type of stimuli; moreover, in some cases, compensating mechanisms override the absence of CCR5 expression and function. Noteworthy, CCR5 may exert a far more important role in the immune response than in immune cells traffic regulation [10].

It has been shown that CCR5 expression in stromal cells as well as hematopoietic cells contributes to tumor metastasis. For instance, CCR5 is involved in chondrosarcomas metastasization [11] and oral cancer cells migration [12]. Its expression correlates with multiple myeloma cell growth, bone marrow homing, and osteolysis [13].

Aster and colleagues [14] showed that T-cell ALL is a disease primarily caused by aberrant activation of the NOTCH1 signaling pathway. In this context, expression and function of important chemokine receptors, such as CCR5 and CCR9, are partially controlled by the oncogenic NOTCH1 isoform in T-cell ALL, regulating blast malignant properties and localization of extramedullary infiltrations [15].

Additionally, CCR5 has been related to play a key role in metastasis of aggressive NK-cells leukemia to the liver of patients, contributing to hepatosplenomegaly and hepatic failure [16, 17]. Also, Davies et al. [18] showed that the G allele of rs1799987 polymorphism in* CCR5* was associated with more favorable minimal residual disease status than the A allele when comparing “best” and “worst” risk groups of B-cell ALL, adjusted for prognostic features. Considering the lower activity of* CCR5* promoter in the presence of this polymorphism [19], this evidence indicated that both acquired and host genetics influence response to cancer therapy. Thus, it is plausible that CCR5 might play a role in ALL pathogenesis and prognosis.

It is known that the polymorphism rs333 in* CCR5*, a common 32-base pair deletion (Δ32), causes truncation and loss of this receptor on lymphoid cell surface, with complete retention in the endoplasmic reticulum within homozygous or diminished expression in heterozygous genotype [20]. The* CCR5* studies have demonstrated the importance of Δ32 mutation, particularly in the susceptibility to HIV infection [21], since CCR5 is a coreceptor in the primary stage of infection that is essential for the AIDS onset [22].

Our research group has reported polymorphic allelic variants related to the immune system and tumor development in different cancer types. Nevertheless, there are no data relating* CCR5*/Δ32 polymorphism to ALL population. In this context, the present work analyzed rs333 polymorphism of* CCR5* in ALL patients from the southern region of Brazil.

## 2. Materials and Methods

### 2.1. Human Subjects

Following approval from the Human Ethics Committee of the State University of Londrina, Paraná, Brazil (CAAE number 171231134.0000.5231), inclusion of the individuals to the study was conditioned by an obtained written informed consent form from parents regarding the use of their children and adolescents blood samples. Seventy-nine ALL patients were enrolled and diagnostic criteria were based on the guidelines proposed by Hematology Department of the University Hospital of Londrina. Recurrence risk status of ALL patients was evaluated through the GBTLI Protocol (Brazilian Group of Childhood Leukemia Treatment Protocol-99) which is based on the Cancer Therapy Evaluation Program, proposed by the National Cancer Institute, and takes into account age at diagnosis, leukocyte count, immunophenotyping, involvement of tissues other than bone marrow, and responsiveness to treatment. The control group is comprised of 80 healthy individuals free of neoplasia, matched by age and gender.

### 2.2. Genomic DNA Extraction

Genomic DNA was extracted from whole blood by Biopur Mini Spin Plus Kit (Biometrix Diagnostica, Curitiba, Brazil), according to the manufacturer's instructions. DNA was eluted in 50 *μ*L of milliQ water and quantified by NanoDrop 2000c spectrophotometer (Thermo Fisher Scientific Inc., Wilmington, USA) at a wavelength of 260/280 nm. Final preparation was stored at −20°C and used as templates in polymerase chain reactions (PCR).

### 2.3. Optimization of PCR for CCR5

The method of genotyping (rs333) was optimized in the Laboratory of Study and Application of DNA Polymorphisms of the State University of Londrina using genomic DNA and specific primers for* CCR5*:* Primer sense*: 5′ ACC AGA TCT CAA AAA GAA 3′ and* Primer antisense*: 5′ CAT GAT GGT GAA GAT AAG CCT CA 3′ (GenBank accession AF009962). Genotyping of CCR5 was determined by PCR-SSP. The samples were amplified using 1.25 units of Taq polymerase (Invitrogen, Carlsbad, USA) in a Mastercycler Gradient Thermal Cycler (Eppendorf, Hamburg, Germany). PCR conditions were: denaturation step at 94°C for 5 min, 35 cycles of 1 min at 94°C, 1 min at 58°C and 1 min at 72°C, and 10 min of elongation at 72°C. PCR products (225 bp or 193 bp) were analyzed on polyacrylamide gel (10%), stained with silver nitrate (AgNO_3_).

### 2.4. Statistical Analysis

Contingency tables and Fisher's exact test were used to calculate differences in genotype distributions and allele frequencies. *P* < 0.05 was considered to indicate a statistically significant difference. Goodness of fit of Hardy-Weinberg equilibrium was tested by calculating the expected frequencies of each genotype and comparing them with the observed value using a chi-square test.

## 3. Results

The distribution of* CCR5* alleles in both ALL and control groups was in accordance with the assumption of Hardy-Weinberg equilibrium (*P* > 0,05). The mean age of controls and ALL patients was 10.8 years ± 5.65 and 8.7 years ± 6.20, respectively, all of whom were predominantly from Caucasoid population, due to European colonization.

**Figure 1:**
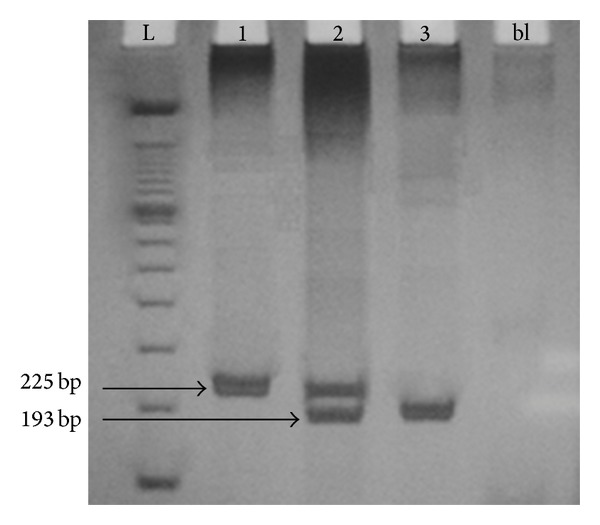
*CCR5* genotype profile. The PCR products were detected using silver staining method after polyacrylamide gel electrophoresis.* Lane* L: DNA Ladder 100 bp;* lane* 1: CCR5 wild-type homozygous genotype (CCR5/CCR5, 225 bp);* lane* 2: heterozygous genotype (CCR5/Δ32, 225 bp, and 193 bp); and* lane* 3: variant allele homozygous genotype (Δ32/Δ32, 193 bp); bl represents blank reaction (without DNA).

The patients and controls were matched for sex and gender, although there was a modest higher frequency of males in ALL group (53.16%) than in controls (48.75%). Fifty (63.29%) ALL patients were classified in high recurrence risk group and 29 (36.71%) in low recurrence risk group. The possible observed genotypes for* CCR5* rs333 polymorphism are shown in Figure 1.

Genotyping results did not show any homozygous individuals for Δ32 deletion in both groups. The heterozygous CCR5/Δ32 genotypes were observed in 8.75% (*n* = 7) of controls and 7.5% (*n* = 5) of ALL patients. To determine if there was a statistically significant increased risk of ALL development related to the *CCR*5 genotypes, we conducted logistic regression analysis (Table 1), which showed that individuals with one copy of Δ32 variant allele did not exhibit ALL-associated risk. No statistical difference was observed when allelic frequency of Δ32 at rs333 in ALL patients was associated with control subjects (OR = 0.71; 95%CI = 0.22–2.30; *P* = 0.77).

In addition, we compared the* CCR5* genotype distribution in ALL patients classified in high risk or low risk, according to recurrence status. From five (7.5%) ALL Δ32 carriers, three were classified as high-risk patients. However, association study between both recurrence statuses did not reach statistical significance.

The* CCR5* genotypes distribution in ALL patients and controls was stratified by gender. Subgroup analyses revealed that the effect of gender was not significantly different among* CCR5* genotypes (female ALL versus female control, OR = 0.52; 95%CI = 0.09–3.07, and male ALL versus male control, OR = 0.97; 95%CI = 0.18–5.14).

## 4. Discussion

Chemokines and their receptors are key regulators of immune activities and in parallel could play conflicting roles in malignancy. While most combinations of these receptors and chemokines are active in cancer, many findings in the field have emphasized the chemokine CCL5 and its cognate receptor CCR5 [23].

The gene variants of the chemokine and chemokine receptor genes associated with inflammation may be involved in cancer initiation and progression [24]. Considering the remarkable difference in CCR5/Δ32 allele frequency among worldwide populations, we aimed to survey the genetic variations in* CCR5* in ALL patients and control individuals.

The patients' age in this study was the expected for ALL, which is frequent in children and younger patients. Moreover, as previously mentioned, both sample groups were composed predominantly of Caucasian individuals from Southern Brazil. However, due to high degree of miscegenation of Brazilian population and the demand to use genetic markers for correct characterization of individuals [25, 26] in our country, these data have not been explored in relation to the variants analyzed.

Chemokines and chemokine receptors are among factors that may influence ALL progression and localization [15]. A 32-base pair nucleic acid deletion in* CCR5* exists and causes a frameshift mutation in the amino acids comprising the second extracellular loop. This deletion leads to premature truncation of the protein, disabling its ability to translocate to the membrane, impairing expression and ligand binding at the cell surface, and causing membrane receptor deficiency that may influence leukocyte trafficking [27].

Based on this, we hypothesized that the ability of CCR5 to bind its ligands and signal recruitment of pathogenic T cells into target tissues may be impaired, thus imparting ALL protection. Although there were no differences in the frequency of CCR5/CCR5 and CCR5/Δ32 genotypes between patient and control groups, the variant genotype had no effect on the ALL susceptibility.

These results corroborated with studies in different disorders, such as leishmaniasis, breast, laryngeal, thyroid, and brain carcinoma, which also identified no differences in the frequency of these alleles among healthy subjects and patients of Southern Brazilian population [28–30] and worldwide [31–33].

Intensive multiagent chemotherapy regimens and introduction of risk-stratified therapy have substantially improved cure rates for children with ALL. Current risk allocation schemas are imperfect, as some children are classified as lower-risk and treated with less intensive therapy relapse, while others deemed higher-risk are probably overtreated [34]. In this context, genetic polymorphisms in chemokine receptors could predict outcome and be considered an independent risk factor to stratify and allocate therapy in ALL.

More than half of the patients with ALL were classified in higher-risk group, according to the clinical and laboratorial findings at diagnosis, as defined by GBTLI LLA-99 protocol [2]. When the genotype data were analyzed for stratified group of ALL, the results indicated that the presence of Δ32 did not influence this clinical parameter. Similarly, a recent study conducted by our research group has not found association among tumor suppressor* TP53* and chemokine* CXCL12* polymorphisms and ALL recurrence risk status [35].

## 5. Conclusion

The comprehension about cellular and molecular mechanisms of ALL is critically important for the development of new approaches to hematological neoplasia treatment. Although any association of rs333 polymorphism of* CCR5* was verified, we believe that the current research must lead to a better definition of the host-tumor relationship particularly with respect to immunologic response and interrelation of CCR5 and ALL development. Given the sample size of the present case-control association study, strong conclusions are not possible; however, future investigation involving much larger cases may determine the absence of clinical implications for CCR5/Δ32 alleles in relation to ALL pathogenesis.

## Figures and Tables

**Table 1:** Genotype distribution of *CCR5* rs333 polymorphism and recurrence risk status analysis in ALL and control groups.

	Genotypes		OR	95% CI	P*
	CCR5/CCR5	CCR5/Δ32			
Control (80)	73 (91.25%)	7 (8.75%)	0.7	0.21–2.32	0.76
ALL (79)	74 (92.5%)	5 (7.5%)			
High risk (50)	47 (94%)	3 (6%)	0.86	0.13–5.48	1.00
Low risk (29)	27 (93.1%)	2 (6.9%)			

*Fisher's exact test, *P* > 0.05. OR: odds ratio; CI: confidence interval.
